# Early prediction of platelet recovery with immature platelet fraction in patients receiving hematopoietic stem cell transplantation

**DOI:** 10.1007/s00277-024-05951-1

**Published:** 2024-09-03

**Authors:** Tsung-Han Yang, Chun-Kuang Tsai, Hao-Yuan Wang, Po-Shen Ko, Sheng-Hsuan Chien, Ting-An Lin, Wen-Chun Chen, Te-Lin Hsu, Chiu-Mei Yeh, Ching-I Lu, Wan-Jou Lin, Ying-Ju Chen, Chia-Jen Liu, Chun-Yu Liu

**Affiliations:** 1 Institute of Emergency and Critical Care Medicine, National Yang Ming Chiao Tung University, Taipei, Taiwan; 2 Division of Transfusion Medicine, Department of Medicine, Taipei Veterans General Hospital, No. 201 Shipai Road, Sec. 2, Taipei, 11217 Taiwan; 3 Division of Hematology, Department of Medicine, Taipei Veterans General Hospital, Taipei, Taiwan; 4 School of Medicine, National Yang Ming Chiao Tung University, Taipei, Taiwan; 5 Division of Medical Oncology, Department of Oncology, Taipei Veterans General Hospital, No. 201 Shipai Road, Sec. 2, Taipei, 11217 Taiwan; 6 Institute of Public Health, National Yang Ming Chiao Tung University, Taipei, Taiwan; 7 Department of Medicine, Kinmen Hospital, Ministry of Health and Welfare, Kinmen, Taiwan

**Keywords:** Hematopoietic stem cell transplantation, Immature platelet fraction, Platelets, Transfusion, Thrombopoiesis

## Abstract

Hematopoietic stem cell transplantation (HSCT) is pivotal in treating hematologic disorders, yet it poses the risk of post-transplantation pancytopenia. Prophylactic platelet transfusions are often administered to mitigate this risk. Utilizing practical markers, such as immature platelet fraction (IPF), to predict hematopoietic recovery in advance could reduce unnecessary prophylactic transfusions. Our prospective study, involving 53 HSCT patients at Taipei Veterans General Hospital between September 2022 and May 2023, utilized the Sysmex XN analyzer to assess peripheral blood cell parameters. We investigated whether IPF could predict platelet recovery early, determined the optimal cut-off value, and compared platelet usage. Neutrophil and platelet engraftment occurred 10 (median; range: 10–12) and 15 (median; range: 15–18) days post-HSCT. Notably, 71.7% of patients exhibited an IPF increase exceeding 2% before platelet recovery. The optimal cut-off IPF on day 10 for predicting platelet recovery within five days was 2.15% (specificity 0.89, sensitivity 0.65). On average, patients received 3.89 units of post-transplantation platelet transfusion. Our results indicate that IPF serves as a predictive marker for platelet engraftment, peaking before the increase in platelet count. This insight aids clinicians in assessing the need for prophylactic platelet transfusions. Integrating reference IPF values alongside platelet counts enhances the accuracy of evaluating a patient’s hematopoietic recovery status. Anticipating the timing of platelet recovery optimizes blood product usage and mitigates transfusion reaction risks.

## Introduction

Hematopoietic stem cell transplantation (HSCT) is a pivotal therapeutic approach for various hematological disorders, involving the infusion of healthy stem cells to restore hematopoietic function. Notably effective against high-risk and relapse-prone hematopoietic malignancies, HSCT employs stem cells from bone marrow, peripheral blood, or umbilical cord blood [1]. Hematopoietic stem cells can be harvested from three primary sources: bone marrow, peripheral blood, and umbilical cord blood. Currently, peripheral blood stands as the primary source for transplantation. HSCT can be categorized as autologous or allogeneic and offers a curative option for various leukemic diseases and non-malignant conditions like aplastic anemia and severe combined immune deficiency [2].

Leukemia is one of the leading causes of cancer-related mortality in Taiwan, prompting nearly one-tenth of the annually reported 3,000 cases to undergo HSCT as a curative measure [3]. However, post-HSCT complications, encompassing myelosuppression, graft-versus-host disease, and infections, necessitate effective care interventions during the pancytopenia phase [4, 5]. Current clinical practices rely on platelet counts to guide transfusions and assess hematopoietic function recovery. Nevertheless, the absence of a dedicated indicator monitoring this recovery poses a global unmet need that could substantially refine post-transplantation care and improve patient outcomes.

Immature platelets, analogous to reticulocytes for red blood cells, represent the youngest circulating platelets and play a crucial role in platelet regeneration [6]. Recognized as the immature platelet fraction (IPF), these platelets can be stained with thiazole orange dye and differentiated from mature platelets via flow cytometry [7]. IPF is a non-invasive biomarker for assessing platelet recovery kinetics. During platelet production, megakaryocytes release immature platelets into the bloodstream before full maturation, distinguishable by their larger size and higher RNA content. Measurement of IPF via flow cytometry quantifies the percentage of these immature platelets in the total platelet population [8]. One study by Taha et al. highlights that immature platelet counts (IPC) remain unaffected by platelet transfusions, while IPF decreases due to such transfusions [9]. Another study by Marc et al. reveals the stability of IPF in EDTA tubes for up to 24 h, contrasting with its instability after six hours in citrate tubes [10].

Recent research has surged in exploring the diagnostic potential of IPF in various disorders, including immune thrombocytopenia [6, 11–13], sepsis [14–16], hypertensive pregnancy disorders [17, 18], and inflammatory conditions like acute bronchitis and COVID-19 infection [19, 20]. Some studies have suggested that IPC, compared to IPF, exhibits more excellent predictive capability and is consistent in diseases such as lupus, sepsis, and COVID-19 [21–23]. IPF has also emerged as a promising predictor of post-transplantation hematopoietic recovery in patients undergoing HSCT. However, the literature on the predictive value of IPF is conflicting, with some studies reporting that IPF increases earlier than platelet count and others reporting no association between IPF and platelet engraftment. A study by Noreen van et al. found that IPF increased two days earlier than platelet count in 16 autologous HSCT patients, with a cut-off value of 5.3% for predicting platelet engraftment [24]. Another study by Julian et al. found that IPF was five days ahead of platelet count in 35 allogeneic bone marrow transplantation patients [25]. Due to the characteristic early rise in IPF peak compared to platelet engraftment, post-transplantation IPF has the potential to facilitate optimal platelet transfusion [26, 27]. However, Meintker et al. found no association between IPF and platelet engraftment following high-intensity chemotherapy or stem cell transplantation [28]. The discrepancies in the literature may be due to differences in study design, population characteristics, and methods of IPF measurement. Additionally, there is no established cut-off value for IPF as a predictor of platelet recovery. Apart from IPF as a predictor of platelet engraftment, immature reticulocyte fraction (IRF) can also indicate neutrophil engraftment post-transplantation [29].

This study aims to address this gap through a prospective investigation encompassing both autologous and allogeneic HSCT in the Taiwanese population. The primary objective is to determine whether IPF or IPC can reliably predict platelet engraftment and establish an optimal cut-off value. This research holds the potential to refine blood transfusion precision, conserve blood products, and elevate the overall quality of patient care. Existing literature primarily comprises retrospective studies with limited sample sizes, often focusing separately on autologous or allogeneic HSCT. This prospective, integrated study seeks to contribute comprehensive insights into the predictive role of IPF in HSCT, offering valuable guidance for clinical practice.

## Materials and methods

### Study population

This prospective observational study enrolled patients aged ≥ 20 who were scheduled for HSCT at Taipei Veterans General Hospital (TVGH) in Taiwan between September 2022 and May 2023. Both autologous and allogeneic transplantation patients were included in this study. Most donors for allogeneic transplantation came from matched unrelated donors, followed by haploidentical donors. All stem cells were collected from peripheral blood through apheresis. Indications for HSCT included multiple myeloma, acute myeloid leukemia, B-cell acute lymphoblastic leukemia, diffuse large B cell lymphoma, chronic myelomonocytic leukemia, myelodysplastic syndrome, angioimmunoblastic T-cell lymphoma, primary myelofibrosis, severe aplastic anemia, extragonadal germ cell tumors, and primary central nervous system lymphoma. The study was approved by Taipei Veterans General Hospital’s Institutional Review Board (no. 2022-05-008ACF).

### Sample collection

Venous whole-blood samples were collected using K_2_-EDTA vacutainer tubes (BD Diagnostics). Our analysis focused solely on the samples collected at 7 AM during the routine daily blood sampling of patients in the hemato-oncology ward. Samples obtained at other times were not included. All tests were performed within six hours of collection. Patient data were collected from the initiation of the conditioning regimen until the day of both neutrophil and platelet engraftment.

### Sample measurement

All hematology tests were performed on the Sysmex XN-1000 analyzer (Sysmex, Kobe, Japan) at the TVGH hematology laboratory. Two-dimensional scatter plots were generated using data acquired through flow cytometry, utilizing a semiconductor laser. The X-axis depicts the intensity of the side fluorescent light (SFL), while the Y-axis corresponds to the intensity of the forward scattered light (FSC). The neutrophil count is an indicator of bone marrow engraftment, defined as a sustained neutrophil count exceeding 0.5 × 10^9^/L for three consecutive measurements on different days.

The DC sheath flow method is the standard technique for platelet counting and measures the shift in direct current resistance due to platelets passing through the system’s aperture. The optical method utilizes platelet fluorescence by introducing thiazole orange dye and accomplishes platelet counting through fluorescence analysis in the reticulocyte channel. The calculation of the IPF relies on PLT-F due to the differing fluorescence characteristics of immature platelets compared to mature platelets. This variation is primarily linked to the increased RNA content in immature platelets. The reticulocyte channel records event counts and applies heightened gating parameters for FSC and side scatter (SSC) to account for the unique characteristics of immature platelets. The IPF (%) is calculated using the following formula:

$$\:\frac{\text{P}\text{a}\text{r}\text{t}\text{i}\text{c}\text{l}\text{e}\:\text{c}\text{o}\text{u}\text{n}\text{t}\text{s}\:\text{i}\text{n}\:\text{t}\text{h}\text{e}\:\text{I}\text{P}\text{F}\:\text{z}\text{o}\text{n}\text{e}}{\text{P}\text{a}\text{r}\text{t}\text{i}\text{c}\text{l}\text{e}\:\text{c}\text{o}\text{u}\text{n}\text{t}\text{s}\:\text{i}\text{n}\:\text{t}\text{h}\text{e}\:\text{p}\text{l}\text{a}\text{t}\text{e}\text{l}\text{e}\text{t}\:\text{z}\text{o}\text{n}\text{e}}\times\:100$$

 Platelet count recovery is another marker for assessing bone marrow engraftment. Platelet engraftment is defined as a sustained platelet count exceeding 20 × 10^9^/L for seven consecutive results on different days without platelet transfusion.

### Study outcomes

The primary outcome was to examine the correlation between an IPF increase of ≥ 2.0 or 2.5% and platelet engraftment after HSCT, and the secondary outcome was to establish the diagnostic accuracy of IPF in predicting platelet engraftment. Prophylactic platelet transfusion guidelines refer to the “Blood Component Utilization Guidelines of Taipei Veterans General Hospital.” For patients combined with congenital or acquired bone marrow dysfunction, when platelet count is < 20 × 10^9^/L, prophylactic platelet transfusion can be administered to prevent bleeding [30].

### Statistical analysis

The baseline characteristics of HSCT patients are presented as the total number (*n*) and proportion (%) of patients. Quantitative variables were presented using the mean and standard deviation (SD) for datasets exhibiting a relatively symmetrical and normal distribution, while the median and interquartile range were employed for datasets with skewed distributions. After calculating the days to neutrophil and platelet engraftment, as well as the number of days with IPF ≥ 2.0 and 2.5%, the results were presented as medians. The Wilcoxon signed-rank test was used to compare the day of platelet engraftment to the day of IPF ≥ 2.0% or 2.5%. We constructed a receiver operating characteristic (ROC) curve and defined optimal IPF and IPC cut-off values using Youden’s index method to maximize sensitivity and specificity for predicting platelet engraftment probability. All data management and statistical analysis were performed using IBM SPSS statistics version 24 (IBM Corporation, Armonk, NY, USA). All tests were two-sided, with a *p* value < 0.05 indicating statistical significance.

## Results

### Clinical characteristics of the study population

This study prospectively enrolled 55 patients who underwent HSCT at TVGH between September 2022 and May 2023. Two patients were excluded: one died in the early stage on the 13th day after transplantation, and the other refused to sign the consent form during the process. The final study cohort included 53 patients (Table 1). The median age of the study patients was 59, ranging from 51 to 66, and 56.7% of the patients were men. The median BMI of the patients was 23.69, ranging from 21.78 to 26.62. Eastern Cooperative Oncology Group (ECOG) stages 0–I and ≥ II were 83.0% and 17.0%, respectively. Of the patients included in the analysis, 35 underwent autologous HSCT, and 18 underwent allogeneic HSCT. Of the latter, 10 received stem cells from matched unrelated donors (MUD), six from haploidentical donors, and two from matched sibling donors. Eight patients received post-transplant cyclophosphamide (PTCy) as graft-versus-host disease prophylaxis. Stem cells for all patients in this cohort were obtained through peripheral blood stem cell isolation procedures. The primary diagnosis for these patients was multiple myeloma, accounting for 39.6% of cases, followed by acute myeloid leukemia (17.0%). Detailed descriptions of other diseases can be found in Table 1. All patients achieved neutrophil engraftment within 30 days after transplantation, but three patients (5.7%) did not achieve platelet engraftment within the same timeframe. The median platelet counts before the first platelet transfusion following transplantation was 12 × 10^9^/L (10–16). The mean units of platelet transfusion administered to patients within 30 days post-transplantation was 3.89 (SD 4.79). Four transfusion reaction events were reported: three patients experienced skin itch and one’s body temperature rose by one degree Celsius after platelet transfusion.

**Table 1:** Baseline characteristics of hematopoietic stem cell transplantation patients

Characteristics	Total *n* = 53
Sex (male)	29 (54.7%)
Age at transplant, median (IQR)	59 (51–66)
BMI, median (IQR)	23.69 (21.78–26.62)
ECOG	
0–1	44 (83.0%)
≥ 2	9 (17.0%)
HSCT type	
Autologous	35 (66.0%)
Allogeneic	18 (34.0%)
Donor type	
Matched unrelated	10 (55.6%)
Haploidentical	6 (33.3%)
Sibling	2 (11.1%)
PTCy use	8 (44.4%)
Cell source	
Bone marrow	0 (0%)
Peripheral blood	53 (100%)
Disease	
Multiple myeloma	21 (39.6%)
Acute myeloid leukemia	9 (17.0%)
Diffuse large B-cell lymphoma	5 (9.5%)
B-cell acute lymphoblastic leukemia	4 (7.5%)
Primary CNS lymphoma	4 (7.5%)
Angioimmunoblastic T-cell lymphoma	2 (3.8%)
Germ cell tumors	2 (3.8%)
Myelodysplastic syndrome	2 (3.8%)
Others	4 (7.5%)
Neutrophil engraftment	53 (100%)
Platelet engraftment	50 (94.3%)
Pre-transfusion platelet counts (×10^9^/L), median (IQR)	12 (10–16)
Unit of platelet transfusions, mean (SD)	3.89 (4.79)

BMI, body mass index; ECOG, Eastern Cooperative Oncology Group performance; HSCT, hematopoietic stem cell transplantation; PTCy, post-transplant cyclophosphamide

Other diseases: Chronic myelomonocytic leukemia, primary myelofibrosis, primary mediastinal large B-cell lymphoma, severe aplastic anemia

### Neutrophil and platelet engraftment

The median for neutrophil engraftment in the 53 patients (100%) included in this study was 10 days post-transplantation, ranging from 10 to 12. Fifty patients (94.3%) achieved platelet engraftment with a median time of 15 days post-transplantation, ranging from 15 to 18. However, three patients (5.7%) did not achieve platelet engraftment by the criteria of the study, as they required continuous platelet transfusions within 30 days post-transplantation. Among the 34 patients (64.2%) who experienced an increase in IPF values greater than 2.5% post-transplantation, the median number of days to the first occurrence was 11.5, ranging from 10 to 13. Similarly, among the 38 patients (71.7%) who experienced an increase in IPF values greater than 2.0% post-transplantation, the median number of days to the first occurrence was also 11.5, ranging from 10 to 13. Notably, the increase in IPF was observed 3.5 days earlier (*p* < 0.001) than the median number of days required for platelet engraftment (Table 2).

**Table 2:** Hematologic recovery parameters

	%	*P* value	Total, *n* = 53
IPF, minimum	0.4 (0.2–0.6)		53
IPF, maximum	3.4 (2.4–5.5)		53
	Days		
Neutrophil	10 (10–12)		53
Platelet	15 (15–18)		50
IPF ≥ 2.0%	11.5 (10–13)	< 0.001*	38
IPF ≥ 2.5%	11.5 (10–13)	< 0.001**	34

IPF, immature platelet fraction

Data expressed as median (interquartile range)

Time frame for minimum and maximum values of IPF is day 0 to day 35 post transplantation

*Significant difference between platelet engraftment and IPF ≥ 2.0% (Wilcoxon signed-rank test)

**Significant difference between platelet engraftment and IPF ≥ 2.5% (Wilcoxon signed-rank test)

### Measurement of IPF and the correlation of IPF rise and platelet engraftment

**Fig. 1:**
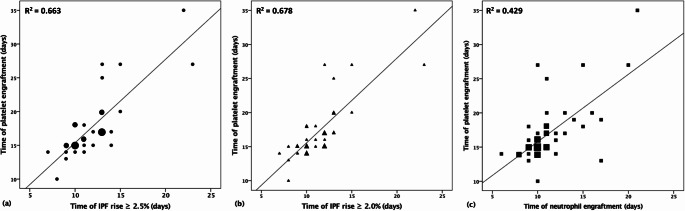
Correlation between engraftment of platelets and IPF rise ≥ 2.5% (**a**), IPF rise ≥ 2.0% (**b**) and engraftment of neutrophils (**c**) in transplant recipients

After transplantation, the patients’ IPF was measured each morning until platelet engraftment. Approximately two-sevenths (28.6%) of the values were missing during the period due to measurements not being taken on weekends. The IPF minimum and maximum value was 0.4% (range from 0.2 to 0.6%) and 3.4% (range from 2.4 to 5.5%), respectively (Table 2). The time frame for data collection extended from post-transplantation day 0 to a maximum of day 35. According to our results, a significant correlation was apparent in each scenario when comparing the duration for IPF increase (≥ 2.0% and ≥ 2.5%) with the time required for platelet engraftment in all patients (Table 3). The strongest correlation was observed when the threshold value for IPF was established at 2.0% (Table 3; Fig. 1b). The correlation between IPF and platelet engraftment is more pronounced than the correlation between IPF and neutrophil engraftment (Fig. 1c).

**Table 3:** Correlation of time of IPF rise (x) with time of platelet engraftment (y)

		Spearman’s ρ	*P* value
IPF ≥ 2.5%	y = 3.01 + 1.24 * x	0.763	< 0.001
IPF ≥ 2.0%	y = 3.11 + 1.24 * x	0.792	< 0.001

IPF, immature platelet fraction

### ROC curve analysis

**Fig. 2:**
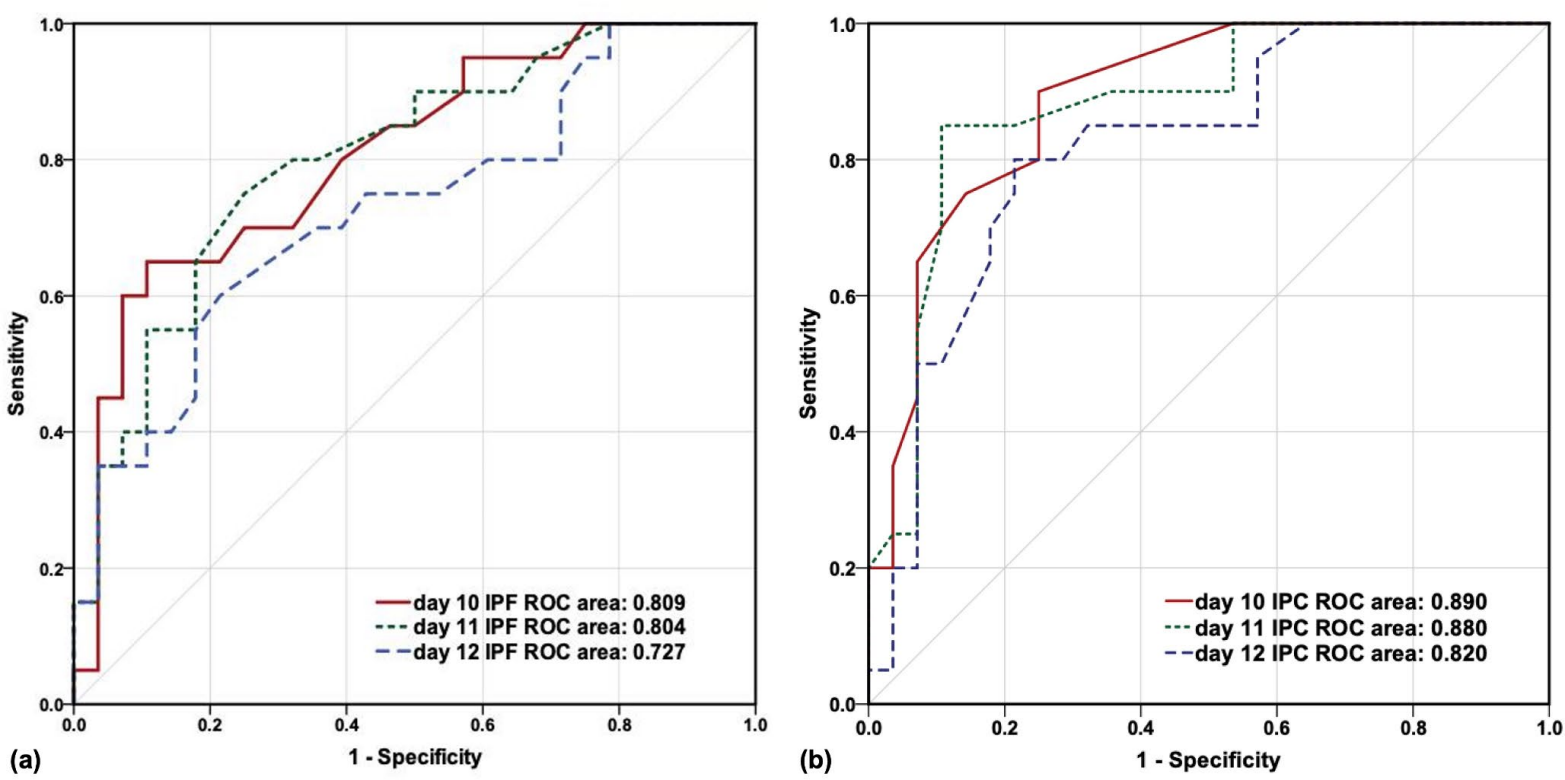
ROC curves and AUCs of immature platelet fraction (**a**) and immature platelet count (**b**) at different days after hematopoietic stem cell transplantation

Based on the median days for platelet engraftment and IPF values greater than 2.0% and 2.5% obtained previously, we analyzed all patients’ IPF values on post-transplantation days 10, 11, and 12 to predict platelet engraftment on day 15 post-transplantation. The ROC analysis of the IPF values on days 10, 11, and 12 resulted in areas under the curve (AUCs) of 0.809, 0.804, and 0.727, respectively. The AUC for day 10 IPF was the highest. The ROC curves are presented in Fig. 2a. According to the Youden index derivation, the optimal cut-off value at day 10 IPF was determined to be 2.15%. Sensitivity, specificity, and positive predictive value were 65.0%, 89.3%, and 85.4%, respectively, for predicting platelet engraftment five days later.

In analyzing another indicator, immature platelet count (IPC), to predict platelet engraftment on day 15 post-transplantation, the ROC analysis revealed AUC values of 0.890, 0.880, and 0.820 for IPC on days 10, 11, and 12, respectively. The IPC on day 10 exhibited the highest AUC (Fig. 2b). Subsequently, utilizing the Youden index analysis, the optimal cut-off value for IPC on day 10 was determined to be 0.55 × 10^9^/L. Sensitivity, specificity, and positive predictive value for predicting successful platelet engraftment five days later were 75.0%, 85.7%, and 84.0%, respectively.

### Recovery of thrombopoiesis following transplantation

**Fig. 3:**
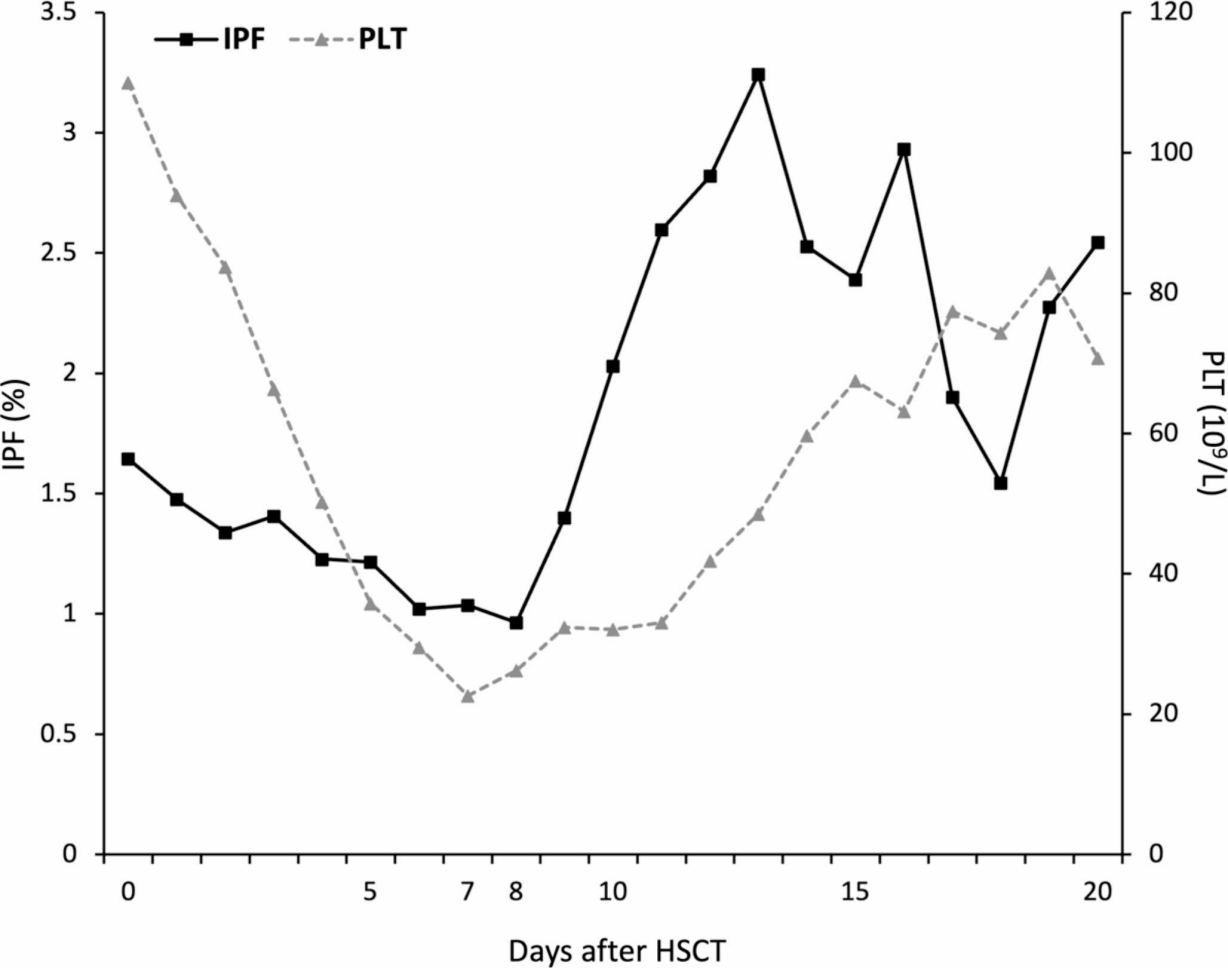
The course of mean values for platelet counts and immature platelet fraction in patients after hematopoietic stem cell transplantation

In all patients, both platelet count and IPF exhibited a gradual decline following the initiation of transplantation, reaching a nadir that endured for varying durations. Figure 3 shows the mean platelet parameters in all patients. Platelet counts and IPF values reached their nadir on post-transplant days 7 and 8, respectively, and subsequently exhibited a gradual upward trend. When IPF reaches 2.0%, the mean platelet count is 32.46 × 10^9^/L (SD 19.36). Of 53 patients, 12 have platelet counts ≤ 20 × 10^9^/L. When IPF reaches 2.5%, the mean platelet count is 35.83 × 10^9^/L (SD 18.93), with only seven patients having platelet counts ≤ 20 × 10^9^/L. As illustrated in Fig. 3, the recovery of IPF values preceded and occurred more rapidly than that of platelet counts.

## Discussion

After HSCT, patients commonly experience a period of pancytopenia, characterized by a decrease in all blood cell counts. During this phase, clinicians must determine the minimum platelet count that patients should maintain while awaiting hematopoietic recovery [31]. Although platelet transfusion is a relatively safe prophylactic treatment, recent shortages of blood products resulting from the pandemic have raised concerns [32, 33]. Additionally, frequent platelet transfusions may lead to transfusion reactions and other complications. Moreover, the guideline on platelet transfusion from the *British Journal of Haematology* advised against administering prophylactic platelet transfusions to asymptomatic patients undergoing autologous stem cell transplantation who do not exhibit signs of bleeding [34]. As opposed to a prophylactic approach, the therapeutic platelet transfusion strategy significantly reduces the quantity and cost of platelets required for transfusion in patients, with no discernible difference in patient prognostic outcomes [35]. Prior research suggests that frequent platelet transfusions may suppress thrombopoiesis [36]. Therefore, identifying early predictors of platelet recovery is imperative for blood bank inventory management and patient care. The measurement of IPF is a convenient and rapid method that can address the dilemma encountered during the waiting period for recovery [37].

Before defining the IPF cut-off value, we observed an IPF increase of more than 2% or 2.5% (median on day 11.5) occurred before platelet engraftment (median on day 15). This suggests that post-transplant IPF is an indicator of impending platelet recovery. We established a cut-off value of 2.15% for IPF on the tenth-day post-transplantation to predict the successful recovery of platelets on the fifteenth day, with a specificity of 0.89. A study in the Netherlands found that IPF increased two days earlier than platelet count in 16 autologous HSCT patients, with a cut-off value of 5.3% for predicting platelet recovery [24]. However, a German study found that IPF cannot predict the recovery of platelets after transplantation or chemotherapy [28]. These discrepancies may stem from variations in study populations and the differences in the biological reference range of the IPF across different ethnicities [38, 39]. Therefore, it is imperative to establish cut-off values for different population groups. Another benefit of measuring IPF is its capability to support prompt clinical decision-making during instances of delayed hematopoietic recovery, especially for patients at higher risk of transplant failure, such as those who receive insufficient doses of CD34^+^ cell infusions or undergo allogeneic transplantation with certain HLA disparities [40]. By monitoring IPF values, clinicians can easily and closely observe early signs of any hematopoietic changes. Notably, utilizing IPF as a potential indicator of the necessity of platelet transfusion presents pros and cons in clinical practice. The advantage is that when a patient’s platelet count is anticipated to rise in a few days, there is no need for transfusion, which can serve as a basis for prioritizing blood product provision when blood bank inventory is low. The disadvantage is that a high IPF does not necessarily indicate a high platelet count at the moment, potentially underestimating the needs of patients at risk of bleeding or with clinical demands. Consequently, sole reliance on IPF as the determinant for platelet transfusion necessity may be inadequate. Instead, clinicians should comprehensively evaluate each patient’s clinical condition to make informed decisions regarding platelet transfusion requirements. However, based on our results, we could not establish a relationship between IPF values and transplant failure, as only three out of the 53 patients in this study did not achieve the defined successful platelet engraftment.

This study has several limitations. First, it is a single-center study, which limits its generalizability. Future studies should involve multiple centers to provide a more comprehensive perspective. Second, there were missing values in the IPF measurements. To address this challenge, we investigated the effects of storing EDTA specimens at room temperature and 4 °C for 24 and 48 h, respectively, before retesting IPF values. However, we observed substantial variations in IPF values, irrespective of storage conditions, with no discernible pattern. Therefore, we imputed missing data with interpolation during critical post-transplantation timeframes (e.g., days 10–15 post-transplantation). However, it is essential to note that this interpolation may not accurately reflect the actual IPF values that would have been obtained if measurements had been conducted on those specific days. Third, although most patients received platelet transfusions during the recovery period, we did not exclude the potential impact of platelet transfusions on IPF values. Previous research has suggested that platelet transfusions may underestimate IPF values [27]. In addition to the advantage of being less affected by platelet transfusion, IPC has shown better AUC performance in other diseases such as thrombotic thrombocytopenic purpura [41]. Therefore, future studies can further investigate not only the impact of platelet transfusion on IPF but also include more patients and observe over a longer period to explore the relationship between IPC and engraftment stability post-transplantation.

In conclusion, our study demonstrates that the readily available and automated IPF value obtained through the Sysmex XN-1000 analyzer is a promising early predictor of platelet hematopoietic recovery following HSCT. IPF results can be simultaneously obtained with complete blood counts from peripheral blood, making it a convenient and accessible tool for clinical use. Therefore, in addition to providing platelet counts, including reference IPF values can more accurately reflect a patient’s current hematopoietic recovery status. Anticipating when a patient’s platelet is likely to recover not only optimizes the utilization of blood products but also reduces the risk of transfusion reactions in patients.

## Data Availability

No datasets were generated or analysed during the current study.
